# Induction chemotherapy reduces extracellular heat shock protein 72 levels, inflammation, lipoperoxidation and changes insulin sensitivity in children and adolescents newly diagnosed with acute lymphoblastic leukemia

**DOI:** 10.18632/oncotarget.25609

**Published:** 2018-06-19

**Authors:** Ana Paula Trussardi Fayh, Camila de Carvalho Gomes, Helena Trevisan Schroeder, Carlos Henrique de Lemos Muller, Telma Maria de Araújo Moura Lemos, Mauricio Krause

**Affiliations:** ^1^ Departamento de Nutrição, Centro de Ciências da Saúde, Universidade Federal do Rio Grande do Norte, Natal, RN, Brazil; ^2^ Laboratory of Inflammation, Metabolism and Exercise Research (LAPIMEX) and Laboratory of Cellular Physiology, Departamento de Fisiologia, Instituto de Ciências Básicas da Saúde, Universidade Federal do Rio Grande do Sul, Porto Alegre, RS, Brazil; ^3^ Departamento de Análises Clínicas e Toxicológicas, Centro de Ciências da Saúde, Universidade Federal do Rio Grande do Norte, Natal, RN, Brazil

**Keywords:** acute lymphoblastic leukemia, chemotherapy, eHSP72, oxidative stress, glycaemia and inflammation

## Abstract

**Background:**

Acute lymphoblastic leukemia (ALL) is associated with higher levels of pro-inflammatory cytokines and oxidative stress. Recently, the levels of extracellular heat shock protein 72 (eHSP72) were found to be elevated in ALL, and its elevation associated with poor prognosis. Therefore, considering the possible role of eHSP72 as a modulator of the immunological system and metabolism, the aim of this study was to describe the response of eHSP72 to the induction phase of chemotherapy, along with metabolic, inflammatory and oxidative stress markers, in children and adolescents newly diagnosed with ALL.

**Methods:**

Nineteen patients were recruited and analysed before and after the induction phase of chemotherapy (with 28 days of duration). Blood samples were taken for the analysis of C-reactive protein (CRP), levels of lipoperoxidation, insulin (and HOMA-IR), cortisol, glucose, lipid profile and eHSP72.

**Results:**

We found that induction phase of chemotherapy leads to a drop in glucose levels (from 101.79±19 to 75.8±9.7 mg/dL), improvements on inflammation (CRP levels, p<0.01) and oxidative stress (TBARS levels, p<0.01), reduction on eHSP72 (p=0.03) and improved insulin sensitivity (HOMA-IR, p=0.02).

**Conclusion:**

Our results indicate that eHSP72 may have an immune and metabolic role and could be used as a marker of the treatment success and metabolic changes in children with ALL.

## INTRODUCTION

Cancer development is often associated with chronic states of inflammation, hormonal unbalance, hyperinsulinemia and insulin resistance [1, 2]. There are growing evidences showing the role of low-grade inflammation in the pathogenesis of cancers, tumor growth and metastasis. Interestingly, several differences on the involvement of inflammation in the pathogenesis of adult-type cancers and the typical malignancies of childhood were identified [3]. Nevertheless, the role of inflammation in pediatric malignancies remains not well understood.

Acute lymphoblastic leukemia (ALL) is the most common pediatric malignancy, but it can occur at any age. ALL is a disorder caused by an abnormal expression of genes, which are usually a result of chromosomal translocations [4]. Leukemic patients present significantly higher proinflammatory cytokines, such as tumor necrosis factor alpha (TNF-alpha) and interleukin 6 (IL-6), at the onset of disease and before cancer therapies, confirming the presence of an inflammatory status in these patients [5]. Increased TNF-α signalling, for example, is responsible for several changes in target cells and tissues that may cause insulin resistance, skeletal muscle atrophy and oxidative stress [6]. Unbalanced redox state has been also implicated in carcinogenesis, as it seems to play an important role in cell proliferation and cell signalling regulation [7]. Accumulation of reactive oxygen species (ROS) leads to genomic instability, and it is known that carcinogenesis is induced by oxidative DNA damage and mutations [8].

In addition to the well characterized pro-inflammatory cytokines involved on ALL, an emerging role for the heat shock proteins (HSPs) family has been suggested. HSPs are considered part of a family of proteins known as “stress proteins” since their expression is induced by a wide range of stressors, such as oxidative stress [9], thermal stress [10], ischemia [11], exercise [9], metabolic stress [12] and many others. The 72 kDa member of the 70 kDa family of heat shock proteins, HSP72 (encoded by the HSPA1A gene in humans), is inducible during cell stress. As molecular chaperones, the intracellular HSP72 proteins (iHSP72) can interact with other proteins (unfolded, in non-native state and/or stress-denatured conformations) to avoid inappropriate interactions, formation of protein aggregates and degradation of damaged proteins, as well as helping the correct refolding of nascent proteins [13]. Other functions include protein translocation [14], anti-apoptosis [15] and anti-inflammatory responses [16, 17]. More recently, the HSP roles have been expanded to include control of cell signaling [18], modulation of immune response [19], and modulation of chronic disease conditions [20] such as diabetes, obesity and insulin resistance [21, 22].

A vital aspect regarding HSP72 physiology is its versatility to induce antagonistic actions, depending on the location of the protein [23]. For example, iHSP72 exerts a powerful anti-inflammatory effect, while eHSP72 has the opposite role, inducing the activation of several pro-inflammatory pathways. In fact, chronic exposure to eHSP72 [97] induces the activation of several pro-inflammatory pathways via the binding to membrane Toll-like receptors, although eHSP72-peptides have also been shown to act as anti-inflammatory and immunosuppressive after internalization and antigen processing [24]. In fact, elevated levels of iHSP72 in cancer cells is associated with tumor cell survival, proliferation, differentiation, invasion and metastasis [25, 26] and, for this reason, iHSP72 is a target in hematological malignancies therapies [27]. Interestingly, clinical correlations has been found between eHSP72 (plasma) and acute leukaemia [28, 29], where increased concentrations of eHSP72 indicates poor prognosis associated with a tendency toward shorter survival.

Therefore, considering the possible role of eHSP72 not only as a marker of ALL development, but also as a modulator of the immunological system and metabolism, the aim of this study was to describe the response of eHSP72 to the induction phase of chemotherapy in children and adolescents newly diagnosed with ALL. In addition, we attempt to understand the relation of eHSP72 with metabolic, inflammatory and oxidative stress parameters.

## RESULTS

Patients had age between one and fifteen years-old, most were under 10 years of age (children), and ten (52.6%) were male. Table 1 shows the anthropometric and general characteristics of the patients. In general, patients had adequate nutritional status and a typical anemia found in newly diagnosed ALL patients. No patient had malnutrition (anthropometric status) at the time of diagnosis and at the end of the treatment, but there was a statistically significant weight loss during the induction phase of chemotherapy (-10.2 ± 2.5%, to 23 kg (12.4 – 34.5) for 19.6 kg (11.6 – 33.2), p<0.01). Serum levels of electrolytes were within the normal range.

**Table 1:** General characteristics of patients at diagnostic

	Male (n=10)	Female (n=9)	Total (n=19)
Age (months)	93 (29 - 133)	59 (41 - 112)	90 (33 - 129)
Children	06 (60%)	07 (77.8%)	13 (68.4%)
Adolescents	04 (40%)	02 (22.2%)	06 (31.6%)
Weight (kg)	26.4 (12.3 - 37)	18.7 (13.8 – 31,4)	23 (12.4 – 34.5)
Height (m)	1.23 ± 0.29	1.17 ± 0.25	1.20 ± 0.26
Body Mass Index/age			
Underweight	-	01 (11.1%)	01 (5.3%)
Normal	09 (90%)	07 (77.8%)	16 (84.2%)
At risk for overweight, overweight or obesity	01 (10%)	01 (11.1%)	02 (10.5%)
Hemoglobin (g/dL)	7.71 ± 1.99	9.33 ± 1.48	8.48 ± 1.91
Hematocrit (%)	22.86 ± 12.57	27.89 ± 4.55	25.24 ± 5.82
Sodium (mmol/L)	136.8 ± 1.87	137.25 ± 2.96	137.0 ± 2.35
Potassium (mmol/L)	4.24 ± 0.37	4.22 ± 0.34	4.23 ± 0.35
Calcium (mg/dL)	9.14 ± 0.68	9,06 ± 0.76	9.11 ± 0.70

Data are presented in mean ± standard deviation or number of individuals (frequency). No differences between genders (p > 0.05) for all variables.

The results of routine biochemical analysis in the first phase of the treatment are presented in Table 2. Positive changes in blood cell count were observed, along with increases in hepatic enzymes and urea, with a reduction in serum sodium. Surprisingly, glucose levels were reduced by ∼25% after induction phase. However, plasma insulin was not affected indicating, based on HOMA-IR results, increased insulin sensitivity (HOMA-IR).

**Table 2:** Biochemical analysis before and after the induction phase of chemotherapy

	Before	After	P value
Haemoglobin (g/dL)	8.79 ± 1.85	9.77 ± 0.55	0.07^*^
Hematocrit (%)	26.10 ± 5.72	29.2 ±2.09	0.06^*^
Leucocytes (mm^3^)	6,300 (3,500 – 18,600)	2,150 (1,075 – 3,350)	**0.02#**
Lymphocytes (%)	83 (73 - 91)	70 (61.5 - 81)	0.07#
Platelets (mm^3^)	68,000 (33,000 – 131,000)	200,000 (151,250 – 295,750)	**0.01#**
GOT (U/L)	28.73 ± 4.35	53.13 ± 4.35	0.03^*^
GPT (U/L)	19.07 ± 8.01	61.00 ± 59.64	0.04^*^
Urea (mg/dL)	15 (12 – 22)	35 (22 - 38)	**0.01#**
Sodium (mmol/L)	137.0 ± 2.24	133.07 ± 2.43	< 0.01^*^
Potassium (mmol/L)	4.27 ± 0.37	4.55 ± 0.98	0.29^*^
Calcium (mg/dL)	9.16 ± 0.76	9.04 ± 0.27	0.67^*^
Glucose (mg/dL)	101.79 ± 19.30	75.84 ± 9.78	< 0.01^*^
Insulin (UI/mL)	8.5 (3.7 – 12.4)	6.65 (4.8 – 8.6)	0.27#
HOMA-IR	2.13 (1.00 – 2.88)	1.28 (0.97 – 1.65)	**0.02#**

Data are presented in mean ± standard deviation or median and interquartile ranges. P value for paired t-test (^*^) or Wilcoxon test (#). Statistically significant differences were in bold. No differences between genders (p < 0.05) for all variables.

Results from CRP (as a marker of inflammation) and TBARS (a marker for lipoperoxidation) indicates that induction phase (for 28 days) decrease the levels of inflammation and oxidative stress (CRP decreases from 7.3 (3.3 – 2.9) mg/dL to 2.5 (1.3 – 4.4) mg/dL, and TBARS decreases from 3.83 ± 0.80 nmol/mL to 2.34 ± 0.63 nmol/mL, p<0.05 for both). Regarding the levels of eHSP72, when analyzing the remaining patients (from 19 subjects only 13 samples were successfully obtained and analyzed) we found a similar pattern, where eHSP72 tends to decrease from 1.40 (1.00 – 3.39) ng/mL to 1.23 (0.69 – 1.97) ng/mL, however no statistical significance was reached. Saying that, we decided to analyze the individual responses to see the general behavior of eHSP72 to chemotherapy. Interestingly, 70% of the subjects decreased their plasma levels of eHSP72. For this reason, we decided to go on a new type of analysis (multiple imputation data - described on the methods) to see if the results would indicate a significant statistical difference, when considering the missing data. In fact, the results from this analysis have indicated that eHSP72 can significantly decrease after the induction phase of chemotherapy.

**Figure 1:**
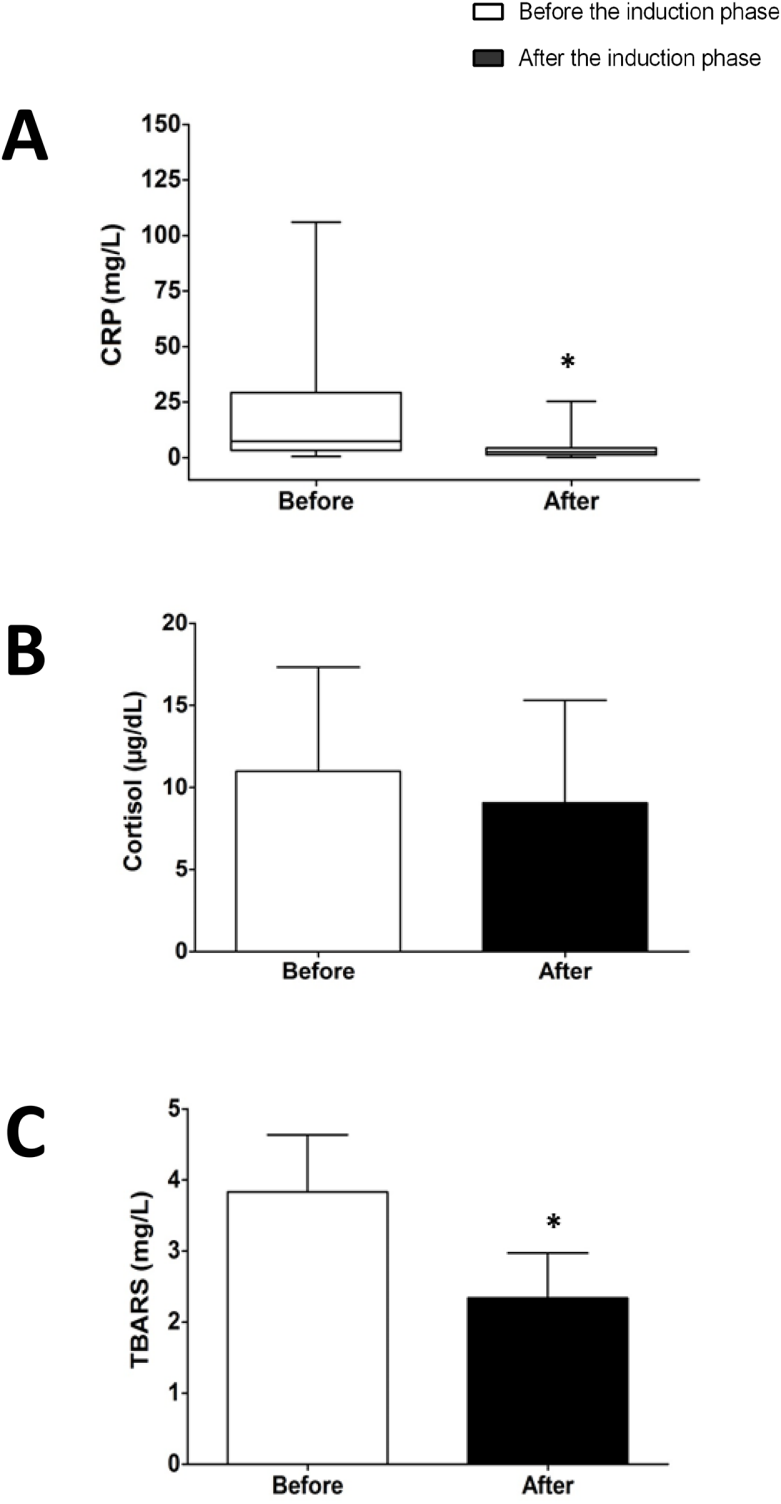
Results of C-reactive protein (CRP) **(A)**, cortisol **(B)** and thiobarbituric acid reactive substances (TBARS) **(C)** form ALL patients before chemotherapy - “before induction”) and at the end of the induction phase (28 days after - “after induction”). Data are expressed in mean ± standard deviation (compared with paired t-test or median and interquartile ranges (compared with Wilcoxon test). ^*^ p< 0.05.

Cortisol levels was the only biochemical marker of stress that did not suffer significant reduction with treatment (from 11.92 ± 5.99 μg/dL to 9.25 ± 5.94 μg/dL, p > 0.27), demonstrating the efficiency of induction phase of chemotherapy to reduce oxidative stress and inflammation in a relatively short period of time (Figure 1). Interestingly, we found an interesting positive correlation (spearman) between eHSP72 and CRP (p= 0.005/r=0.481) and eHSP72 vs. leucocyte number (cells that can export HSP72 to the blood) (p=0.039/r=0.351).

## DISCUSSION

Herein, we have shown that induction phase of chemotherapy induces several changes over metabolic, inflammatory and oxidative stress parameters. Of most interest, glucose levels dropped significantly, insulin sensitivity increased, oxidative stress and inflammation reduced. This was followed by a concomitant decline on eHSP72 levels. The lower eHSP72 levels after induction phase, based on previous studies, predicts a better prognosis, however, more than just a marker, eHSP72 drop may be involved with the better inflammatory, metabolic and oxidative profile of the ALL patients.

Despite the induction phase being attributed to induce a transient hyperglycaemia in ALL patients [30], in our hands, induction phase induced a significant fall on glucose levels. The effects of chemotherapy on glucose levels is assigned, at least in part, to the effects of corticosteroids (either prednisone or dexamethasone) and asparaginase. The first, by increasing glycogenolysis and gluconeogenesis and, the second, by reducting insulin secretion (via inhibition of cAMP levels) [30, 31]. Previous work have identified age >10 years, female, obesity, family history of diabetes, and Down syndrome as risk factors for the development of transient hyperglycaemia [32]. This is important to consider, since our group was composed mostly of children (average of 6.4 ± 4.1 yo). Thus, glycemia response to induction therapy may differ between very young children to adolescents. In fact, hypoglycemia is not unusual in children with ALL in response to induction chemotherapy. Case reports and clinical trials have shown that, for children of lower age, cases of hypoglycemia are more common than hyperglycemia [33, 34].

Metabolically, the different types of leukemia's can present completely distinct profiles. Recently, metabolic alterations associated with two subtypes of acute leukemia, ALL and acute myeloid leukemia (AML), were investigated in the serum of these patients and compared with a healthy control group, using ^1^H NMR (nuclear magnetic resonance) spectroscopy [35]. Remarkably, compared with controls, ALL and AML patients showed serum metabolic differences involving atypical metabolism pathways including glycolysis, TCA cycle, lipoprotein metabolism and fatty acid metabolism. Of interest, ALL patients exhibits higher glucose levels than controls and AML subjects, prior to any treatment, suggesting that ALL patients had elevated secondary metabolic processes for constant supply of glucose as compared to AML and controls. The higher levels of lactate and low levels of other glucogenic amino acids including alanine, glutamine, histidine, lysine, valine, and proline in ALL patients’ serum further supported this change [35].

Following that, the question that arises is: if transient hyperglycemia may be induced by the use of corticosteroids and asparaginase during the course of chemotherapy, what may be the cause of hyperglycemia in ALL patients observed prior to any therapy? The answer may be connected with the increased sympathetic tone that occurs in this condition [36]. Heart rate variability (HRV), for example, depends of autonomic function (balance between sympathetic and parasympathetic tone). Interestingly, newly diagnosed patients with leukemia, present decreased HRV, which reflects sympathetic dominance in acute leukemia [36]. Considering the metabolic effects of the sympathetic nervous system over metabolic function, it is expected that pathways that lead to hyperglycemia would be activated. In addition to the well known metabolic sympathetic effect, this system is now characterized by inducing (activate) changes over the immune system (Th1 polarization). Indeed, sympathetic activity can induce, in white blood cells, a direct monocyte/macrophage stimulation via β-adrenergic-dependent activation of nuclear factor-kB (NF-kB) biochemical pathways. These are then activated via an Ras/ERK cascade, with concomitant rise in NADPH oxidase activity and generation of reactive oxygen species. In turn, NF-kB activation induces production and release of pro-inflammatory mediators, including TNF-α, which may mediate further monocytes/macrophages activation in an autocrine fashion. This was previously demonstrated by studies from our laboratory where a single bout of moderate exercise (that activates sympathetic system) stimulates macrophage function, increasing phagocytic capacity, production of hydrogen peroxide and nitric oxide (NO˙) through the nuclear factor kappa B activation [37, 38].

Moreover, children with ALL often present fever. Curiously, the inflammatory response involved in fever occurs even in the absence of infection. The levels of TNF-α, IL-6, IL8 chemoattractant protein-1 (MCP-1) and IL-10 are higher in patients with ALL when compared to the control children. Moreover, the levels of the T helper 1 (interferon-γ and IL-12) cytokines are higher in patients with ALL while transforming growth factor β is lower in ALL. These results indicate that the circulating levels of cytokines are elevated in patients with newly diagnosed ALL without apparent infection, reflecting a strong and deregulated inflammatory state in this disease, with a Th1-polarization profile [39]. Since the sympathetic nervous system is involved on the activation of this polarization, the increased tone of this system may explain this immune behavior in ALL.

Many other factors are suggested to modulate the immune function, e.g., the release of HSP72 (by hepatocytes and immune cells) and also the activation of sympathetic corticotropin-releasing hormone (CRH)–histamine axis, both dependent on catecholamine action. The production of eHSP72, for example, is mediated by a noradrenergic stimulation via of α1 receptors in the liver, the main organ responsible for its release, but also by immune cells [40, 41]. The eHSP72 plays an important role in promoting the phagocytic activity and production of mediators of the inflammatory response via TLR2 and TLR4 (Toll-like receptors) [40, 41]. Thus, higher sympathetic tone can lead to increased eHSP72 release by hepatocytes and immune cells that may explain, at least in part, the high levels of this protein in ALL. In addition, eHSP72 can induce a vicious cycle with other inflammatory cytokines, activating a Th1 profile and culminating into a forward feedback that induce more inflammation in these patients. In fact, chronically increased levels of eHSP72 have been associated with inflammation [42], heart failure mortality, sarcopenia [43] and cognitive decline [44]. In addition, increased levels of eHSP72 are positively correlated with traditional inflammatory markers such as IL-2/IL-10 ratio and others [45]. Thus, reductions of eHSP72 are a desirable change in inflammatory-associated conditions such as obesity, diabetes, aging and LLA.

Low-grade inflammation is well known to induce insulin resistance. Therefore, increased cytokine levels, along with eHSP72 may induce to oxidative stress pathways activation in plasma and several tissues that, in the case of skeletal muscle, may blunt the insulin signaling. Recent data from our laboratory showed that the levels of intracellular HSP72 (iHSP72) is positively correlated with insulin signaling (sensitivity), while the eHSP72 may induce inflammation and insulin resistance [40, 41, 42]. Considering our present data, we found that after induction phase, levels of inflammation, eHSP72 and oxidative stress (lipoperoxidation), significantly decreased. This was followed by the simultaneous drop in insulin resistance (measured by HOMA-IR). Thus, lower inflammation and eHSP72 may reduce the effects of these factors over insulin sensitivity and, for this reason, blood glucose levels dropped consistently. It is important to consider that asparaginase can lead to changes in insulin secretion, however, in our hands, no changes in insulin levels were found, indicating that glucose uptake was increased by improvements on insulin sensitivity. Furthermore, as previously mentioned, a high expression of iHSP72 can result in increased insulin sensitivity in several tissues [41]. Considering this, recent research have shown that glucocorticoid treatment in ALL models induced a better heat shock response (the pathway involved on the HSP72 expression through activation of HSF-1 [41]), thus increasing iHSP72 levels [46]. If it is the case, higher iHSP72 and lower eHSP72, will lead to increased insulin sensitivity and then glucose uptake, in accordance with our findings. Indeed, eHSP72/iHSP72 ratio was suggested to be determinant on insulin sensitivity [40, 41, 42, 47].

**Figure 2:**
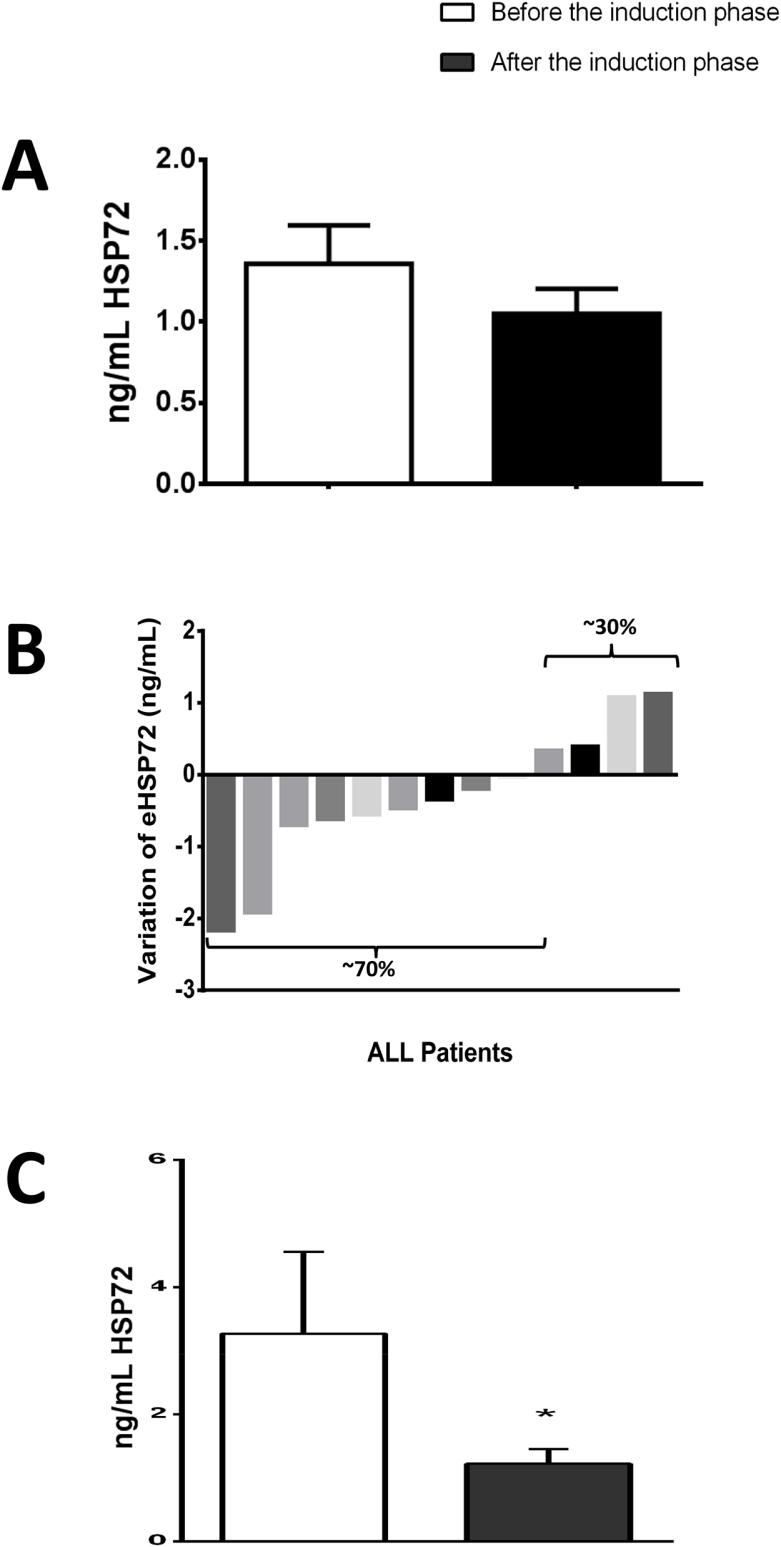
Results of extracellular heat shock protein 70 (eHSP72), form ALL patients before chemotherapy - “before induction”) and at the end of the induction phase (28 days after - “after induction”) **(A)** Results using raw data of individuals who completed the induction phase (from 19 subjects only 13 samples were successfully obtained and analyzed). **(B)** Individual responses to the induction phase (13 subjects: 70% decreased eHSP72 levels and only 30% increased). **(C)** Results after multiple imputation of data (described in the methods). Data are expressed in mean ± standard deviation (compared with paired t-test or median and interquartile ranges (compared with Wilcoxon test). ^*^ p< 0.05.

Summarizing our data, we found that induction phase of chemotherapy in lower age children leads to a drop in glucose levels, improvements on inflammation and oxidative stress markers, reduction on eHSP72 and improved insulin sensitivity (please see Figure 1). Considering our results, we suggest a hypothetical model to explain the differences we found and how these variables are connect among themselves prior and after the induction phase (Figure 2). The proposed mechanismand our results are valid only for the first phase of chemotherapy (induction), and they may change on the time-course of the treatment. In addition, the age must be considered, since glycemic responses may vary between children and adolescents.

**Figure 3:**
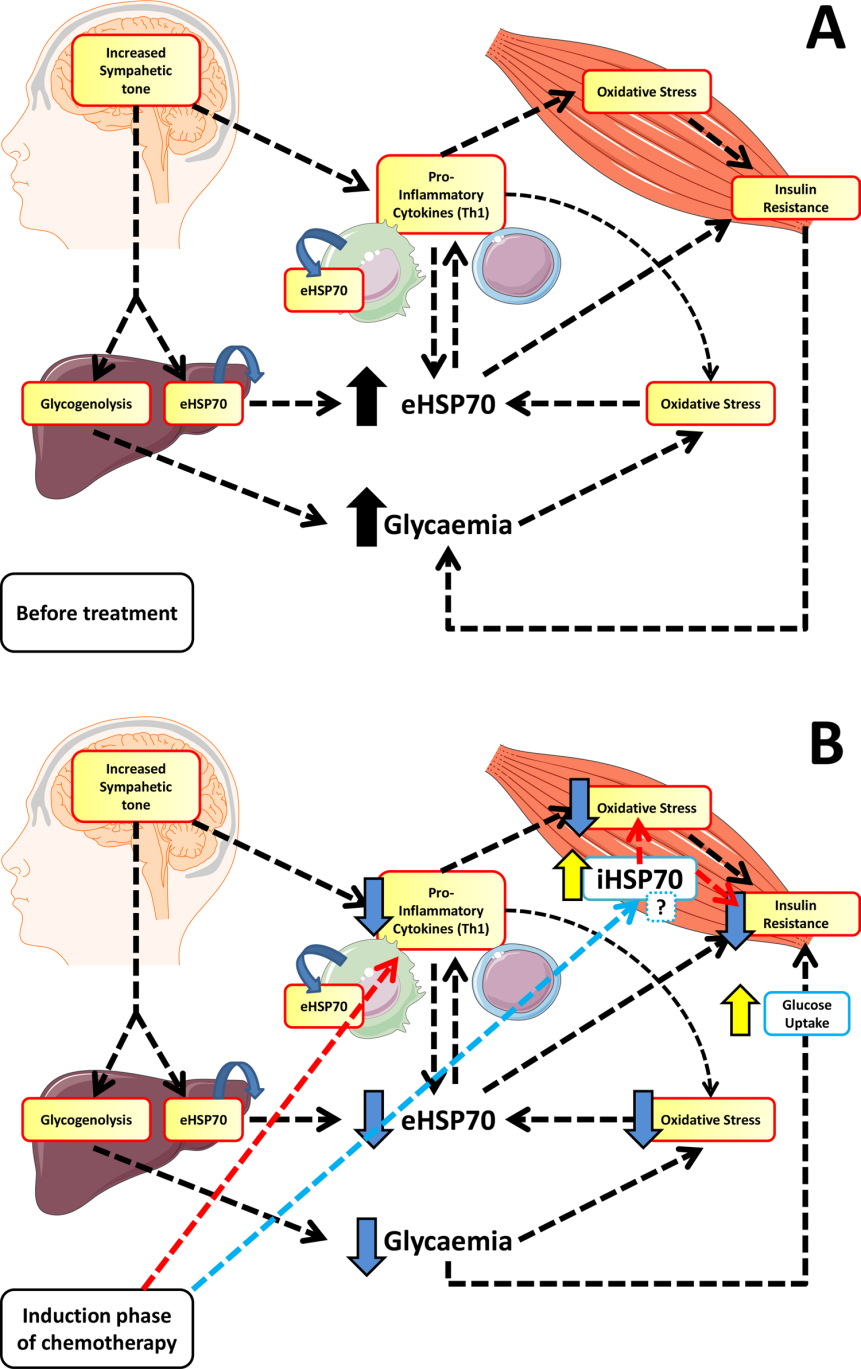
Proposed hypothetical model for the role of eHSP72 in ALL before and after induction phase of chemotherapy The higher sympathetic tone found in ALL children will lead to glycogenolysis (increasing glycaemia) and activation of immune system (Th1 polarization), inducing pro-inflammatory pathways activation and eHSP72 release. eHSP72, along with other cytokines and their stimulation on the ROS/RNS production will induce skeletal (and other tissues) insulin resistance, that together, explains hyperglycemia prior to the treatment **(A)**. Induction drugs used on chemotherapy will decrease inflammation and oxidative stress, that will reduce eHSP72 release. Lower eHSP72 and iHSP72 (changing the eHSP72/iHSP72 ratio) will improve insulin signaling resulting on glucose uptake, thus reducing glycaemia in this first therapy phase **(B)**.

Briefly, the higher sympathetic tone found in ALL children will lead to glycogenolysis (increasing glycaemia) and activation of immune system, inducing pro-inflammatory pathways activation and eHSP72 release. eHSP72, along with other cytokines and their stimulation on the ROS/RNS production will induce skeletal (and other tissues) insulin resistance, that together may explain hyperglycemia prior to the treatment. Induction drugs used on chemotherapy will decrease inflammation (as confirmed by the lower CRP levels, Figure 1), and oxidative stress (reduction on lipoperoxidation, Figure 1), which will reduce eHSP72 release (Figure 3). Lower eHSP72 (as measured) and iHSP72 (not confirmed) will improve insulin signaling resulting on glucose uptake, thus reducing glycaemia in this first therapy phase (lower glucose, Table 2). The suggested mechanism is limited, of course, since not all variables were measured, but partially supported by the positive correlations found between eHSP72, inflammation and leukocyte number. These correlations are not merely mathematical, they have an important biological significance. As the immune system becomes highly activated, the increased inflammatory molecules can stimulates the release of other signaling molecules such as CRP and eHSP72 (also inflammatory) [28, 39, 40]. With more inflammatory cytokines, more inflammation is produced, in a positive feedback loop mechanism. Chemotherapy leads to the reduction of the peripheral blood mononuclear cells (PBMC) number, reducing the release of inflammatory molecules and, as a consequence, decreasing the levels of eHSP72, CRP and other inflammatory markers.

It is important to consider all limitations of this study. The small number of people recruited, and the fact that the disease resulted in considerable losses during the intervention, is a strong limitation. In addition, we only investigated one marker of inflammation and lipoperoxidation. In the future, new markers should be included to have a better comprehension of the effects of the induction phase of chemotherapy. Finally, a caution must be taken when interpreting this result, since that data imputation in small cohorts could mislead conclusions. Despite of that, this results may be valuable for generating new insights and further research in larger studies, even considering that the population studied is very special and quite scarce, particularly, at this stage of chemotherapy.

## MATERIALS AND METHODS

### Participants

This was an observational study in which were included children and adolescents newly diagnosed with ALL in the Pediatric Oncology Department of two hospitals of the city of Natal (Rio Grande do Norte, Brazil) between March and December 2015. Exclusion criteria were metastasis, Down´s syndrome, hypothyroidism, presence of other chronic disease or having started chemotherapy. In total, nineteen consecutively patients were eligible for inclusion. Patients were evaluated in the beginning (before chemotherapy - “before induction” and at the end of the induction phase (28 days after - “after induction”). The goal of induction chemotherapy is to achieve a remission. Independent of risk, children received following chemotherapy drugs: L-asparaginase, methotrexate, prednisone, vincristine and daunorubicin. Ethical approval was obtained from Ethics Committee of the Federal University of Rio Grande do Norte (protocol 976.388).

### Anthropometric and characterization of the sample

Body weight was measured using a Filizola^®^ weighting scale and recorded to the nearest 0.1 kg. Standing height was measured using a calibrated stadiometer for infants (Filizola^®^), and recorded to the nearest 0.1 cm. Body mass index-for-age were calculated according to World Health Organization [48].

### General biochemistry

Venous blood samples were obtained during the morning hours after an overnight fast. Glycaemia, blood cell count, hematocrit, leukocytes, lymphocytes and platelet counts, plasma urea, glutamic oxalacetic transaminase, glutamic pyruvic transaminase, sodium, potassium and calcium were determined by colorimetric enzymatic assay. All measurements were performed at the hospital as part of the routine in the clinical treatment.

### Inflammatory, metabolic and oxidative stress markers

C-reactive protein (CRP) was determined by nephelometry (Boehringer, Germany). Insulin (UI/mL) and cortisol (μg/dL) were analyzed by chemiluminescence (Active Insulin ELISA^®^, Assay Designs Cortisol ELISA^®^ kit, Labtest). Insulin resistance was calculated by the homeostasis model assessment of insulin resistance (HOMA-IR) and β-cell function by using HOMA-β [49]. For the thiobarbituric acid reactive substances assay, a marker for membrane lipoperoxidation, acetic acid was added to the plasma to precipitate proteins and to acidify samples. This mixture was then centrifuged (1000 g/3 min). Thiobarbituric acid (0.67 % w/v) was added to the reaction medium, and tubes were boiled (100°C) for 1 hour. Absorbance was read at 535 nm in a spectrophotometer, and the results were expressed as nmol/mL of plasma [50]. A highly sensitive, enzyme-linked immunosorbent assay (EIA) method (EKS-715 Stressgen, Victoria, BC, Canada) was used to quantify the levels of plasma HSP72 protein as previously described [42]. Absorbance was measured at 450 nm, and a standard curve constructed from known dilutions of HSP72 protein to allow quantitative assessment of HSP72 concentration. Quantification was made using a microplate reader (Multiskan Go, Thermo Scientific, Waltham, EUA). The intra- and inter-assay CV ranged between 4.5 and 7 %.

### Statistical analyses

Data were analyzed by SPSS, v.22 (IBM, Armonk, NY). *Shapiro-Wilk* normality test was applied previously to all analyses. For the descriptive analysis of continuous variables, means ± standard error or medians (interquartile interval) were calculated as appropriate, and compared with independent t-test between sexes. There were missing data for eHSP72. Therefore, methods of multiple imputation data were applied, which follow three main steps: a) imputation of missing data to obtain complete databases; b) estimation based on *n* complete databases, and c) a combination of methods. Data were assumed to be missing at random [51]. Results are presented and discussed under both, with and without imputation of missing values. Comparison of anthropometric, general biochemistry, inflammation and oxidative stress markers in the different moments were compared with paired t-test or Wilcoxon test. Spearman’s rank order correlation coefficient (rs) was used to determine correlations between eHSP72, CRP and other blood variables. Alpha level was set at p<0.05.

## CONCLUSIONS

We found that induction phase of chemotherapy, in lower age children, lead to a drop in glucose levels, improvements on inflammation and oxidative stress markers, reduction on eHSP72 and improved insulin sensitivity. Our results indicate a possible key involvement of eHSP72 as an immune and metabolic controller that may be used as a marker of the treatment success and metabolic changes. However, the pattern of change in our variables (eHSP72, CRP, glucose, etc) needs to be measured, during and after the other stages of the disease and chemotherapy treatment, and with a larger patient number.

## Abbreviations

ALL: Acute lymphoblastic leukemia
AML: Acute myeloid leukemia
CRP: C-reactive protein
eHSP72: extracellular heat shock protein 70
iHSP72: intracellular HSP72 proteins
HOMA-IR: Homeostasis model assessment of insulin resistance
HRV: Heart rate variability
IL-6: interleukin 6
NF-kB: Nuclear factor-kB (NF-kB)
ROS: Reactive oxygen species
TBARS: thiobarbituric acid reactive substances
TNF-α: Tumor necrosis factor alpha

## References

[1] Tabung FK, Fung TT, Chavarro JE, Smith-Warner SA, Willett WC, Giovannucci EL (2017). Associations between adherence to the World Cancer Research Fund/American Institute for Cancer Research cancer prevention recommendations and biomarkers of inflammation, hormonal, and insulin response. Int J Cancer.

[2] Weiser MA, Cabanillas ME, Konopleva M, Thomas DA, Pierce SA, Escalante CP, Kantarjian HM, O’Brien SM (2004). Relation between the duration of remission and hyperglycemia during induction chemotherapy for acute lymphocytic leukemia with a hyperfractionated cyclophosphamide, vincristine, doxorubicin, and dexamethasone/methotrexate-cytarabine regimen. Cancer.

[3] Morgenstern DA, Anderson J (2012). Inflammation: what role in pediatric cancer?. Pediatr Blood Cancer.

[4] Mrózek K, Heerema NA, Bloomfield CD (2004). Cytogenetics in acute leukemia. Blood Rev.

[5] Giordano P, Molinari AC, Del Vecchio GC, Saracco P, Russo G, Altomare M, Perutelli P, Crescenzio N, Santoro N, Marchetti M, De Mattia D, Falanga A (2010). Prospective study of hemostatic alterations in children with acute lymphoblastic leukemia. Am J Hematol.

[6] Jaqueline Santos Moreira Leite VF, Mauricio Krause, Paulo Ivo Homem de Bittencourt (2016). Physiological regulation of the heat shock response by glutamine: implications for chronic low-grade inflammatory diseases in age-related conditions. Nutrire.

[7] Imbesi S, Musolino C, Allegra A, Saija A, Morabito F, Calapai G, Gangemi S (2013). Oxidative stress in oncohematologic diseases: an update. Expert Rev Hematol.

[8] De Marco F, Bucaj E, Foppoli C, Fiorini A, Blarzino C, Filipi K, Giorgi A, Schininà ME, Di Domenico F, Coccia R, Butterfield DA, Perluigi M (2012). Oxidative stress in HPV-driven viral carcinogenesis: redox proteomics analysis of HPV-16 dysplastic and neoplastic tissues. PLoS One.

[9] Krause MS, Oliveira LP, Silveira EM, Vianna DR, Rossato JS, Almeida BS, Rodrigues MF, Fernandes AJ, Costa JA, Curi R, de Bittencourt PI (2007). MRP1/GS-X pump ATPase expression: is this the explanation for the cytoprotection of the heart against oxidative stress-induced redox imbalance in comparison to skeletal muscle cells?. Cell Biochem Funct.

[10] Yang XM, Baxter GF, Heads RJ, Yellon DM, Downey JM, Cohen MV (1996). Infarct limitation of the second window of protection in a conscious rabbit model. Cardiovasc Res.

[11] Richard V, Kaeffer N, Thuillez C (1996). Delayed protection of the ischemic heart—from pathophysiology to therapeutic applications. Fundam Clin Pharmacol.

[12] Beckmann RP, Lovett M, Welch WJ (1992). Examining the function and regulation of hsp 70 in cells subjected to metabolic stress. J Cell Biol.

[13] Madden LA, Sandström ME, Lovell RJ, McNaughton L (2008). Inducible heat shock protein 70 and its role in preconditioning and exercise. Amino Acids.

[14] Chirico WJ, Waters MG, Blobel G (1988). 70K heat shock related proteins stimulate protein translocation into microsomes. Nature.

[15] Garrido C, Gurbuxani S, Ravagnan L, Kroemer G (2001). Heat shock proteins: endogenous modulators of apoptotic cell death. Biochem Biophys Res Commun.

[16] Homem de Bittencourt PI, Lagranha DJ, Maslinkiewicz A, Senna SM, Tavares AM, Baldissera LP, Janner DR, Peralta JS, Bock PM, Gutierrez LL, Scola G, Heck TG, Krause MS (2007). LipoCardium: endothelium-directed cyclopentenone prostaglandin-based liposome formulation that completely reverses atherosclerotic lesions. Atherosclerosis.

[17] Ianaro A, Ialenti A, Maffia P, Di Meglio P, Di Rosa M, Santoro MG (2003). Anti-inflammatory activity of 15-deoxy-delta12,14-PGJ2 and 2-cyclopenten-1-one: role of the heat shock response. Mol Pharmacol.

[18] Calderwood SK, Mambula SS, Gray PJ, Theriault JR (2007). Extracellular heat shock proteins in cell signaling. FEBS Lett.

[19] Johnson JD, Fleshner M (2006). Releasing signals, secretory pathways, and immune function of endogenous extracellular heat shock protein 72. J Leukoc Biol.

[20] Kampinga HH, Henning RH, van Gelder IC, Brundel BJ (2007). Beat shock proteins and atrial fibrillation. Cell Stress Chaperones.

[21] Chung J, Nguyen AK, Henstridge DC, Holmes AG, Chan MH, Mesa JL, Lancaster GI, Southgate RJ, Bruce CR, Duffy SJ, Horvath I, Mestril R, Watt MJ (2008). HSP72 protects against obesity-induced insulin resistance. Proc Natl Acad Sci USA.

[22] Krause M, Rodrigues-Krause JC (2011). Extracellular heat shock proteins (eHSP70) in exercise: possible targets outside the immune system and their role for neurodegenerative disorders treatment. Med Hypotheses.

[23] Rodrigues-Krause J, Krause M, O’Hagan C, De Vito G, Boreham C, Murphy C, Newsholme P, Colleran G (2012). Divergence of intracellular and extracellular HSP72 in type 2 diabetes: does fat matter?. Cell Stress Chaperones.

[24] Borges TJ, Wieten L, van Herwijnen MJ, Broere F, van der Zee R, Bonorino C, van Eden W (2012). The anti-inflammatory mechanisms of Hsp70. Front Immunol.

[25] Ciocca DR, Calderwood SK (2005). Heat shock proteins in cancer: diagnostic, prognostic, predictive, and treatment implications. Cell Stress Chaperones.

[26] Mjahed H, Girodon F, Fontenay M, Garrido C (2012). Heat shock proteins in hematopoietic malignancies. Exp Cell Res.

[27] Kliková K, Pilchova I, Stefanikova A, Hatok J, Dobrota D, Racay P (2016). The Role of Heat Shock Proteins in Leukemia. Klin Onkol.

[28] Yeh CH, Tseng R, Hannah A, Estrov Z, Estey E, Kantarjian H, Albitar M (2010). Clinical correlation of circulating heat shock protein 70 in acute leukemia. Leuk Res.

[29] Yeh CH, Tseng R, Zhang Z, Cortes J, O’Brien S, Giles F, Hannah A, Estrov Z, Keating M, Kantarjian H, Albitar M (2009). Circulating heat shock protein 70 and progression in patients with chronic myeloid leukemia. Leuk Res.

[30] Lowas SR, Marks D, Malempati S (2009). Prevalence of transient hyperglycemia during induction chemotherapy for pediatric acute lymphoblastic leukemia. Pediatr Blood Cancer.

[31] Pou JM, Cervera T, Perez A, Ortiz MA, Arroyo JA (1991). Effect of L-asparaginase on insulin secretion from isolated rat islets of Langerhans. Horm Res.

[32] Baillargeon J, Langevin AM, Mullins J, Ferry RJ, DeAngulo G, Thomas PJ, Estrada J, Pitney A, Pollock BH (2005). Transient hyperglycemia in Hispanic children with acute lymphoblastic leukemia. Pediatr Blood Cancer.

[33] Panigrahi M, Swain TR, Jena RK, Panigrahi A (2016). L-asparaginase-induced abnormality in plasma glucose level in patients of acute lymphoblastic leukemia admitted to a tertiary care hospital of Odisha. Indian J Pharmacol.

[34] Tanaka R, Osumi T, Miharu M, Ishii T, Hasegawa T, Takahashi T, Shimada H (2012). Hypoglycemia associated with L-asparaginase in acute lymphoblastic leukemia treatment: a case report. Exp Hematol Oncol.

[35] Musharraf SG, Siddiqui AJ, Shamsi T, Choudhary MI, Rahman AU (2016). Serum metabonomics of acute leukemia using nuclear magnetic resonance spectroscopy. Sci Rep.

[36] Nevruz O, Yokusoglu M, Uzun M, Demirkol S, Avcu F, Baysan O, Koz C, Cetin T, Sag C, Ural AU, Isik E (2007). Cardiac autonomic functions are altered in patients with acute leukemia, assessed by heart rate variability. Tohoku J Exp Med.

[37] da Silva Rossato J, Krause M, Fernandes AJ, Fernandes JR, Seibt IL, Rech A, Homem de Bittencourt PI (2014). Role of alpha- and beta-adrenoreceptors in rat monocyte/macrophage function at rest and acute exercise. J Physiol Biochem.

[38] Silveira EM, Rodrigues MF, Krause MS, Vianna DR, Almeida BS, Rossato JS, Oliveira LP, Curi R, de Bittencourt PI (2007). Acute exercise stimulates macrophage function: possible role of NF-kappaB pathways. Cell Biochem Funct.

[39] Pérez-Figueroa E, Sánchez-Cuaxospa M, Martínez-Soto KA, Sánchez-Zauco N, Medina-Sansón A, Jiménez-Hernández E, Torres-Nava JR, Félix-Castro JM, Gómez A, Ortega E, Maldonado-Bernal C (2016). Strong inflammatory response and Th1-polarization profile in children with acute lymphoblastic leukemia without apparent infection. Oncol Rep.

[40] Krause M, Bock PM, Takahashi HK, Homem De Bittencourt PI, Newsholme P (2015). The regulatory roles of NADPH oxidase, intra- and extra-cellular HSP70 in pancreatic islet function, dysfunction and diabetes. Clin Sci (Lond).

[41] Krause M, Heck TG, Bittencourt A, Scomazzon SP, Newsholme P, Curi R, Homem de Bittencourt PI (2015). The chaperone balance hypothesis: the importance of the extracellular to intracellular HSP70 ratio to inflammation-driven type 2 diabetes, the effect of exercise, and the implications for clinical management. Mediators Inflamm.

[42] Krause M, Keane K, Rodrigues-Krause J, Crognale D, Egan B, De Vito G, Murphy C, Newsholme P (2014). Elevated levels of extracellular heat-shock protein 72 (eHSP72) are positively correlated with insulin resistance in vivo and cause pancreatic β-cell dysfunction and death in vitro. Clin Sci (Lond).

[43] Ogawa K, Kim HK, Shimizu T, Abe S, Shiga Y, Calderwood SK (2012). Plasma heat shock protein 72 as a biomarker of sarcopenia in elderly people. Cell Stress Chaperones.

[44] Son SJ, Lee KS, Chung JH, Chang KJ, Roh HW, Kim SH, Jin T, Back JH, Kim HJ, Lee Y, Choi SH, Noh JS, Lim KY (2015). Increased plasma levels of heat shock protein 70 associated with subsequent clinical conversion to mild cognitive impairment in cognitively healthy elderly. PLoS One.

[45] Heck TG, Scomazzon SP, Nunes PR, Schöler CM, da Silva GS, Bittencourt A, Faccioni-Heuser MC, Krause M, Bazotte RB, Curi R, Homem de Bittencourt PI (2017). Acute exercise boosts cell proliferation and the heat shock response in lymphocytes: correlation with cytokine production and extracellular-to-intracellular HSP70 ratio. Cell Stress Chaperones.

[46] Hernández-Espinosa D, Miñano A, Ordóñez A, Mota R, Martínez-Martínez I, Vicente V, Corral J (2009). Dexamethasone induces a heat-stress response that ameliorates the conformational consequences on antithrombin of L-asparaginase treatment. J Thromb Haemost.

[47] Krause M, Ludwig MS, Heck TG, Takahashi HK (2015). Heat shock proteins and heat therapy for type 2 diabetes: pros and cons. Curr Opin Clin Nutr Metab Care.

[48] de Onis M, Onyango A, Borghi E, Siyam A, Blössner M, Lutter C, WHO Multicentre Growth Reference Study Group (2012). Worldwide implementation of the WHO Child Growth Standards. Public Health Nutr.

[49] Krause M, Rodrigues-Krause J, O’Hagan C, Medlow P, Davison G, Susta D, Boreham C, Newsholme P, O’Donnell M, Murphy C, De Vito G (2014). The effects of aerobic exercise training at two different intensities in obesity and type 2 diabetes: implications for oxidative stress, low-grade inflammation and nitric oxide production. Eur J Appl Physiol.

[50] Bock PM, Krause M, Schroeder HT, Hahn GF, Takahashi HK, Schöler CM, Nicoletti G, Neto LD, Rodrigues MI, Bruxel MA, Homem de Bittencourt PI (2016). Oral supplementations with L-glutamine or L-alanyl-L-glutamine do not change metabolic alterations induced by long-term high-fat diet in the B6.129F2/J mouse model of insulin resistance. Mol Cell Biochem.

[51] Akl EA, Shawwa K, Kahale LA, Agoritsas T, Brignardello-Petersen R, Busse JW, Carrasco-Labra A, Ebrahim S, Johnston BC, Neumann I, Sola I, Sun X, Vandvik P (2015). Reporting missing participant data in randomised trials: systematic survey of the methodological literature and a proposed guide. BMJ Open.

